# Diet and Exercise in Cancer Metabolism

**DOI:** 10.1158/2159-8290.CD-22-0096

**Published:** 2022-09-05

**Authors:** Jason W. Locasale

**Affiliations:** 1 Department of Pharmacology and Cancer Biology, Duke University School of Medicine, Durham, North Carolina.

## Abstract

Diet and exercise are modifiable lifestyle factors known to have a major influence on metabolism. Clinical practice addresses diseases of altered metabolism such as diabetes or hypertension by altering these factors. Despite enormous public interest, there are limited defined diet and exercise regimens for patients with cancer. Nevertheless, the molecular basis of cancer has converged over the past 15 years on an essential role for altered metabolism in cancer. However, our understanding of the molecular mechanisms that underlie the impact of diet and exercise on cancer metabolism is in its very early stages. In this perspective, I propose conceptual frameworks for understanding the consequences of diet and exercise on cancer cell metabolism and tumor biology and also highlight recent developments. By advancing our mechanistic understanding, I will discuss actionable ways that such interventions could eventually reach the mainstay of both medical oncology and cancer control and prevention.

## INTRODUCTION

Nature and nurture are important in shaping the status of a biological system. Both the genetic makeup and the environment that interacts with the living system are highly relevant to all complex diseases and health statuses. For metabolism—broadly defined as the collection of chemical reactions sourced from the diet that contributes to life—genes encode enzymes that carry out the chemistry and the activity of these genes can be influenced by a host of factors, including the genetics related to transcription programs, signaling pathways, and chromatin-modifying enzymes. Cancer biology, benefiting from the enormous advances in technology over the past 50 years, has defined much of the critical genetic makeup of the disease (1). For example, the progression to metastasis or the differential susceptibility to a therapy can sometimes be predicted by the somatic mutations in oncogenes and tumor suppressor genes that have been selected in the pathogenesis of the disease. Concomitantly, there has been a surge of interest in cancer metabolism over the past 15 years in large part due to the finding that many cancer genes such as *KRAS*, *PIK3CA, CMYC*, and *TP53* have substantial effects on tumor metabolism (2). Thus, with genetically defined metabolic programs in tumors, it is now widely accepted that altered metabolism and its functional requirements are major features of cancer biology.

The environmental factors that influence cancer metabolism have been far less studied, but numerous studies have shown they can be equally if not more important than these genetic factors (3). Indeed, the tumor microenvironment that influences tumor metabolism is an active area of research. Ultimately for organismal metabolism, the beginning environmental factor is the diet, which intersects with physical activity and other life exposures. But how diet and exercise propagate within the host to influence cancer metabolism is still largely unknown. Nevertheless, there are major implications for public health, as obesity is thought to cause roughly 5% to 15% of all human cancers.

## PRINCIPLES OF DIET AND MOLECULAR METABOLISM

**Figure 1.**
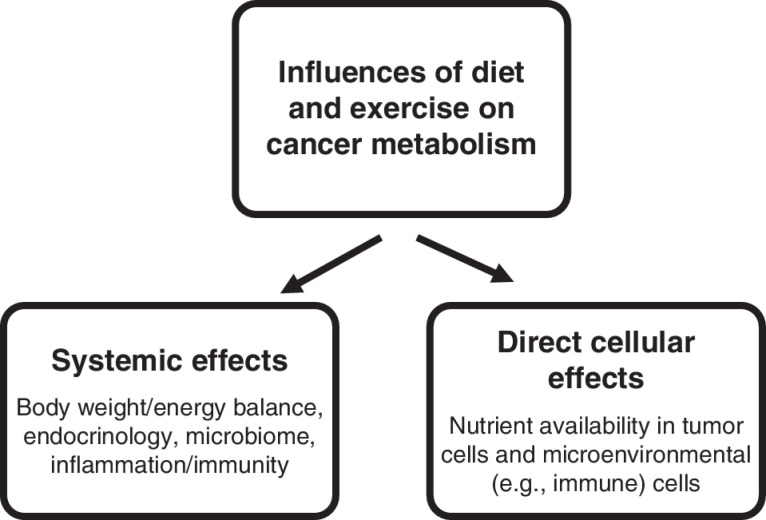
Systemic and direct metabolic influences of diet and exercise on cancer metabolism. Lifestyle factors involving diet and exercise alter tumor metabolism through both systemic influences on organismal physiology (left) and direct effects on cellular metabolism through changes in nutrient availability (right).

Metabolism begins with physical activity, including oxygen consumption, and dietary intake of nutrients. Food is taken up and digested in a number of steps, including absorption and interaction with the gut microbiome, and then transported to and processed by the liver. Nutrients eventually enter circulation where they interact with other peripheral organs. Thus, after vast amounts of systemic regulation including the engagement of countless signaling pathways that affect all of physiology, nutrients eventually reach the site of interest such as the tumor location. Thus, both systemic and direct effects at the cancer site can be important for defining the overall impact of diet on cancer biology (Fig. 1). Furthermore, nutrients are taken up from the vasculature by both the tumor and nontumor cells in the microenvironment. Cells neighboring the tumor cells or other cancer-relevant cells such as an immune infiltrate, through metabolic end products, can provide additional nutrient sources.

A change in diet may or may not influence nutrient availability at the tumor site and in the malignant cells depending on physiologic regulation. However, if a change in nutrient level at the tumor site does occur as a consequence of a change in diet, the mechanism that determines whether this would affect cellular metabolism lies in the biochemical and biophysical properties of nutrient uptake. If a nutrient transporter is present and the nutrient is not in excess with respect to the Michaelis constant (Km) of the given transporter, which is typically the case, then the rate of uptake is either proportional to or a monotonic function of the concentration of the metabolite. Thus, nutrient availability that begins with diet can directly influence metabolic activity or flux in cellular pathways in numerous ways through nutrient uptake before we even begin to consider signal transduction and gene regulation, which also undoubtedly occur.

Signal transduction and nutrient exchange occur across and within each tissue and likely between all cells, and nutrient-sensing pathways detect the presence of changes to nutritional status to engage transcriptional programs and regulate metabolic pathway activity. Perhaps the most well-known case is that of physiologic glucose regulation by insulin. Insulin induces the translocation of the glucose transporter to the plasma membrane in tissues such as muscle, the phosphorylation of hundreds of metabolic enzymes across all tissues, and the transcriptional regulation of metabolic genes through signaling pathways involving kinases such as AKT and mTOR. These and many more factors affect nutrient uptake in cancer-relevant cells, and each is influenced by the diet. Numerous examples abound including glucagon and related peptides that, for example, regulate PKA signaling and in turn affect glycolysis, glycogen production, and gluconeogenesis. Lipids and amino acids have numerous nutrient-sensing mechanisms that influence systemic and cellular metabolism, such as the cholesterol-sensing system involving the transcription factor SREBP and the amino acid–sensing pathways involving mTOR and ATF4. Changes to diet affect metabolism by these and many more signaling pathways (e.g., leptin), in addition to the direct interactions on metabolism that occur through nutrient uptake. Thus, in each case in which diet has been shown to have an effect on cancer metabolism, it is usually not known to what extent the changes in metabolic pathway activity are from nutrient uptake or from systemic interorgan or intercellular signaling. Substantial efforts would need to be undertaken to parse these multiple effects.

Physical activity or exercise also exerts both systemic and cell-autonomous effects on metabolism. Much work has been done to characterize the effects of exercise on skeletal muscle. Induction of both autocrine and paracrine signaling pathways is present. As hallmark features of exercise, programs resulting from the activation of gene transcription and sig­naling pathways including AMPK, PGC1a, mTOR, HIF1a, MAPK, and many others are induced in the muscle during exercise (4). Notably, each of these molecular constituents is a prominent factor in cancer biology and also influences the activity of metabolic pathways. Lesser studied but also likely important are the direct biochemical effects on cellular metabolism that exercise exerts. Changes to oxygen consumption, reactive oxygen species production, and anabolic demands related to tissue regeneration and mechanical stress all affect glucose and mitochondrial metabolism and are acutely and chronically affected by physical activity and exercise through direct changes in electron transport chain activity and possibly mechanotransduction pathways as well. Intriguingly, these all are also well-documented hallmarks of cancer metabolism. Consistently, preliminary studies have shown that exercised mice bearing patient-derived xenografts exhibit alterations to mitochondrial metabolism along with reduced tumor growth (5). More recent studies show that exercise interacts with antitumor immunity (6). Nevertheless, more work is needed to understand the appropriate biomarkers that define the dosing and other features of specificity that are essential in translating this work into humans.

Because there are so many factors that mediate the intersection of diet, exercise, and cancer metabolism, it is perhaps surprising that a specific diet could at all have a consistent effect through nutrient availability at the tumor site. However, studies demonstrate that plasma metabolite levels can be predicted to some extent by diet, with some nutrients being more heavily modifiable than others. Further, there is also a correlation between nutrient levels and the interstitial tissue fluid where the cancer resides, implying that microenvironmental nutrient availability derives in some or large part from metabolites in blood circulation (7). Indeed, in cell culture models, such changes are predicted to have a large effect on cancer metabolism in many cases—sometimes larger than the impact of mutating potent oncogenes that have been thought in general to be the major determinant of altered metabolism. In addition, studies have shown oxygen transport at the tumor site, and by inference other elements of nutrient availability, are influenced by the total body oxygen consumption rate (VO2), suggesting that, in addition to dietary influences, changes to lifestyle in the form of physical activity should also affect cancer metabolism.

## MECHANISMS OF DIET AND EXERCISE AS CANCER THERAPY

Diseases of altered metabolism can be managed by changes to diet and exercise, but the clinical principles that govern these interventions, particularly in cancer, are lacking. In recent years, there has been a surge of interest toward elucidating the mechanisms that link diet to cancer metabolism (8–10). Of note, dietary interventions in preclinical settings have been shown to synergize with chemotherapy, radiotherapy, protein kinase–based targeted therapies, metformin therapy, and even the highly studied immune therapies—all potentially paving the way for personalized dietary interventions in cancer. Here, I highlight some key and recent findings in which new mechanistic insight is beginning to emerge and raises issues for further investigation. Many of these topics have been discussed elsewhere (8–10), so the emphasis here is on new conceptual ideas.

**Figure 2.**
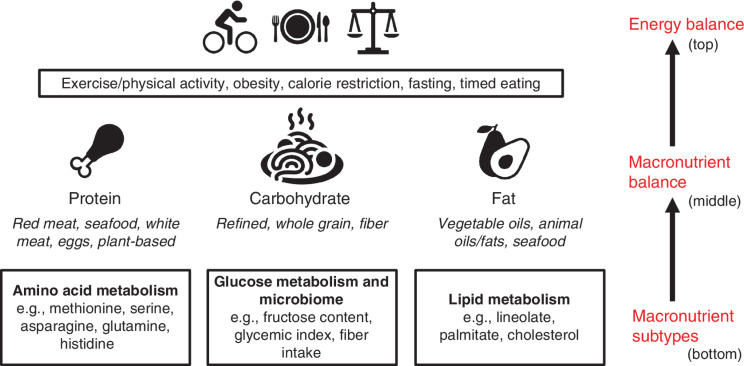
A hierarchical view of diet and exercise and their effects on metabolism. Beginning the hierarchy is energy balance (top). Energy balance is roughly defined as the total calorie intake from food minus energy expenditure, in part through exercise and other energy-dissipating processes such as digestion and thermogenesis. As has been discussed extensively elsewhere and generally speaking, excess energy contributes to obesity. Factors affecting energy balance would include obesity, exercise, timed eating, caloric restriction, and fasting. Next in the hierarchy (middle) is macronutrient balance. This consideration involves the relative dietary intake of the three caloric sources or macronutrients: protein, carbohydrate, and fat. This would be impacted by diets such as ketogenic (or keto), paleo, protein restriction, low carbohydrate, vegan, etc. In light of this hierarchical model, these diets of differential macronutrient composition should first be considered relative to their effects on energy balance. For example, low-carbohydrate diets are often lower in calories, so this should be considered in any conclusions drawn. Finally, the hierarchy considers changes in the dietary intake of the macronutrient subtype (bottom). These diets involve changes to the relative intake of certain types of macronutrients. In lifestyles in the human population, this would involve red meat consumption, veganism, sugary beverage intake, fiber intake, keto, etc. In laboratory studies, this would involve, for example, saturated fat composition and amino acid–depleted diets. In the hierarchical model, the effects these diets have should be considered first in reference to the overall energy balance they may alter and next their effects on macronutrient balance. For example, a diet deprived of serine or restricted of methionine should be considered (i.e., these variables should be controlled) in reference to both its total calories and its total protein. Further, a diet high in fructose should also be considered in reference to both its total calories and its total carbohydrate intake.

A hierarchy of dietary considerations can be conceptualized as a working model in which the lower tiers should be considered in reference to the higher tiers in the ladder (Fig. 2). At the top is the notion of energy balance, which includes features of metabolism such as excess weight gain and storage, physical activity, calorie intake, and dietary timing. In the middle of this hierarchy, macronutrient (i.e., carbohydrates, protein, and fat) intake is considered. Finally, the intake of nutrients within macronutrient categories (e.g., fructose, saturated fat, animal protein) is studied. The hierarchy results from changes to the above category possibly or more likely underlying the effects in question (e.g., tumor growth) due to a change in the lower tiered category. Therefore, each above category should be controlled for when considering the effects seen in the below category. For example, for low-carbohydrate diets, can the weight loss effects be due to changes in total calorie intake? This should be ruled out before concluding that the effect is due to the change in macronutrient balance that occurs when there is differential carbohydrate intake. The same can be said about a diet low in a given type of carbohydrate such as fructose. Can the effects seen be due to changes in overall carbohydrate intake? This should first be ruled out or else one might be considering the effects of the higher tier in the hierarchy.

### Energy Balance

There is the overall energy balance defined as the net energy intake, which includes calorie intake, energy expenditure, as well as energy dissipation and storage. Although it has been challenged, generally speaking, the consensus among scientists and the public is that this is the underlying factor contributing to weight gain and loss (11, 12). Thus, in considering the hierarchy, effects of a diet on health or cancer may be most readily prioritized from this perspective and begin with how the diet affects energy balance. Diets involving fasting, calorie restriction, and timed feeding—for example, changes to eating times over the course of a day or longer—as well as all aspects of exercise and physical activity are of high interest to cancer biology and typically alter this balance. For example, fasting for prolonged periods of time results, in addition to its effects on energy balance through calorie intake, in increased fatty acid oxidation, which changes energy expenditure, as oxidizing lipids uses more energy, albeit at a slower rate, than oxidizing carbohydrates or amino acids. In addition, changes to energy dissipation and storage occur as a result of changes to the timing of eating, even when calorie intake over a given period of time is equal, and also affect energy balance. Thus, generally speaking, diets involving caloric restriction, fasting, or even timed feeding as well as exercise interventions likely result in substantial changes to energy balance; therefore, changes to this balance should be considered when investigating the mechanisms underlying resulting phenotypes. In some settings such as a cyclic fasting regimen during the course of standard cancer therapy, there are substantial effects on tumor metabolism and even antitumor immunity in patients (13). Although it is not known whether changes to energy balance are the cause of the anticancer effects, they must be taken into account (e.g., how much of the effect was due to weight loss) when considering possible mechanisms.

### Obesity and Cancer

Depending on the cohort and type of model analysis, obesity is believed to cause 5% to 15% of all new cancers. According to the NCI, for example, 9.6% of new cancer cases in women are attributable to excess body weight. Thus, after smoking cessation and human papillomavirus vaccination, weight loss intervention becomes the major avenue in cancer prevention. Furthermore, cancer outcome in women and men who are obese is generally worse, which has implications for treatment (14). Although obesity is generally considered to result from aberrant energy balance, there are intense arguments for different sources and causes of this imbalance (11, 12). These include leptin and satiety as well as dysregulated insulin signaling and carbohydrate metabolism. Interestingly, some of these mechanisms, such as altered glucose metabolism and insulin signaling, have been studied in cancer etiology, whereas others such as dysregulated leptin signaling or thermogenesis have been investigated to a lesser extent. Ultimately, despite the intriguing causal link between excess weight and cancer, the mechanisms that link the two and implicate whether this connection can be separated are still poorly understood. Many of the links involve inflammation including T-cell function, as it provides a natural connection because it is so well accepted to contribute to both obesity and cancer pathologies (15, 16). Direct contribution from changes to organismal metabolism including and beyond insulin signaling could be relevant as well. For example, methionine restriction, which has shown defined results in weight loss and cancer (17), can cause tumor regression via cell-autonomous changes to nutrient uptake of methionine (18). Other recent studies have shown that changes to adipose tissue metabolism can affect tumor growth, which appears to have a mechanism more aligned with nutrient exchange across organs and resulting changes to nutrient uptake in the tumor (19). Similar results are observed with calorie restriction or fasting mimicking diets (13, 20), which can cause weight loss. Thus, weight loss and the resulting anticancer mechanisms involved are highly relevant for cancer intervention, but the underlying mechanisms remain to be completely understood.

### Physical Activity and Exercise

As with excess weight and cancer, there is abundant and consistent evidence pointing to the substantial effects of increased physical activity resulting in reductions in cancer incidence. Women who are physically fit, with appropriate caveats to be applied to observational studies, may have up to a 21% reduction in cancer incidence (21). Randomized controlled trials are emerging, and some have shown promising results (22). Thus, in addition to weight loss through diet, exercise is clearly one of the major modifiable factors for reducing cancer mortality. The mechanisms in play are similar to what may be contributing to the role of obesity in cancer. Inflammation has been implicated, and recent studies point to specific effects such as changes to IL15 receptor engagement in CD8^+^ T cells that infiltrate tumors (6). Cell-autonomous changes to metabolism via alterations in mitochondria through changes to oxygen consumption and thus the tricarboxylic acid (TCA) cycle also have been observed to occur concurrently with exercise-induced inhibition of tumor growth in patient-derived xenograft models (5). These mechanisms would also predict that exercise could synergize with immune therapies or drugs that target cancer metabolism. Thus, further mechanistic understanding is key to moving forward with the possibilities of using exercise as a modality in prevention or treatment. For example, how changes to muscle and fat mass status that are influenced by exercise could affect tumor growth is largely unknown. One unresolved matter both clinically and mechanistically is the dosing of the exercise and the extent that this variable should be individualized (23). How exercise dosing affects muscle, other tissues, and tumor tissue/cancer cells in physiologic environments is a major area of interest. For example, in a hypoxic, nutrient-deprived tumor microenvironment, exercise could lead to complex effects on oxygen consumption at the site of cancer cells because the behavior of the mitochondria is generally different in those environments, as is the vasculature and how the response to exercise may change the vasculature at the cancer site. Altogether, much work remains in this emerging area, but it is expected that as with diet in recent years, exercise oncology will merge with current concepts in cancer metabolism and cancer immunology in the coming years.

Nevertheless, it is clear that energy balance is not the only variable relevant to nutrition. Relative macronutrient intake, determined by ratios such as that of carbohydrate to fat intake, are major considerations. Diets high in carbohydrates (e.g., vegan) and high in fat (e.g., ketogenic, or keto) have received widespread popular attention in recent years. Protein amounts, while historically neglected, are also emerging as having important roles in metabolism, but their contents as they relate to carbohydrate and fat intake are far less clear. Finally, intramacronutrient intake (i.e., the type of carbohydrate, protein, and fat) is also of high interest. These concepts are further developed below.

### Protein Intake and Amino Acid Metabolism

Interest in diets that restrict carbohydrates (e.g., low carbohydrate diets/keto or removal of added sugars) or fats (e.g., low saturated fat) is generally very high both in the general public and in clinical investigation (24). Altering protein content has had less of a consideration, and some general perceptions and studies conclude that protein and particularly amino acid intake are relatively constant in diet. However, in the study of cancer metabolism, amino acid metabolizing pathways have emerged as promising drug targets, as they have been shown to be important for cancer proliferation through various functions (8–10, 17). During the Warburg effect (25), the phenomenon of altered glucose metabolism in tumors characterized by increased glucose uptake and fermentation to lactic acid, it was found by using isotope tracing of heavy labeled glucose that tumor cells synthesize increased amounts of the amino acid serine from glucose (26). This occurred through the activity of the enzyme phosphoglycerate dehydrogenase (PHGDH), which is involved in the committed step from glucose to serine synthesis. Serine is biochemically linked to folate and methionine metabolism through the donation of its single carbon side chain to a folate moiety, resulting in the production of glycine. The downstream metabolism is collectively known as one-carbon metabolism and serves a plethora of metabolic functions important for cancer such as nucleotide synthesis, maintenance of redox status, and epi­genomic status. Consequently, studies in xenografts and later in autochthonous, genetically defined mouse tumors were able to show that removing serine and glycine from the diet could induce substantial antitumor responses and even synergize with inhibition of PHGDH, showing that serine availability was the key modifier of the antitumor effects (27, 28).

The amino acid methionine comprises the other key input to one-carbon metabolism (17). Methionine has been shown in some cohorts to be the most variable amino acid in plasma, and estimates using dietary records and quantitative modeling indicated that close to one half of the variation could be explained by diet (29). In addition, dietary methionine restriction is known to confer antiaging and antiobesity properties while leading to specific changes in one carbon–related metabolism. In several laboratory studies involving patient-derived xenografts and genetically engineered mouse models, this same nutritional intervention could interact with some of the common therapies that are coupled to one-carbon metabolism involving radiation and antimetabolite chemotherapy (18). The underlying mechanism appeared to be the enhanced dependence on methionine for coupling to and maintenance of the folate cycle downstream effects when methionine was limiting. Other studies have pointed to sphingolipid metabolism as being relevant to the antitumor properties, which could have some effects on metastasis as well (30, 31). Furthermore, a dietary intervention such as methionine restriction has been shown to achieve a comparable metabolic profile as that in humans eating a plant-based, low-protein diet (32).

Additional studies have shown that other amino acids can induce antitumor effects upon removal, restriction, or increased dietary intake (8–10). Dietary asparagine, whose metabolism is only three reaction steps involving glutamate from the TCA cycle, was found to synergize with the anticancer effects of metformin, a drug targeting mitochondrial metabolism (33). Dietary supplementation with glutamine, whose metabolism can interact with the TCA cycle via two reaction steps involving glutamate, can also exert anticancer metabolism (34). Histidine is another example in which supplementation can lead to synergy with methotrexate, an antifolate agent, as chemotherapy through its coupling to the folate cycle (10). Together, a picture emerges whereby single changes to dietary amino acid content in mice can propagate into the tumor site to influence the metabolic flux related to the amino acid of interest. When that flux is involved in maintaining processes important for cancer proliferation directly or in combination with a therapy of interest, such as its effects on the immune system or on chemo- or radiotherapy, a therapeutic outcome can be achieved. These studies further converge on an important concept that protein content, both in its quantity and quality (i.e., the amino acid content a given type of protein confers), is a dietary variable of interest to cancer and more relevant to disease than currently considered (35). More work is needed, however, in relating these lab studies to the human diet. For example, there is an interesting anticorrelation between serine and methionine (serine is higher in poultry and methionine higher in red meat), but whether this can manifest to specific changes in physiologic metabolism is unclear. Applying metabolomics analysis in more controlled studies of the human diet will be helpful in parsing these effects. Of course, this is not to say that the better studied macronutrients involving glucose and lipid metabolism are not also important, and their relation to cancer and diet is discussed below.

### Carbohydrate Intake and Glucose Metabolism

As has also been shown in the course of studying the Warburg effect, growth factor signaling pathways and transcription factors that are fundamental to oncogenesis directly regulate the activity of glucose, mitochondria, and TCA cycle metabolism. These pathways are connected through numerous mechanisms including the glycolytic intermediate pyruvate and the redox cofactor NAD^+^. The TCA cycle is involved in redox biochemistry, reactive oxygen species production, oxygen consumption, and ATP production, along with anabolic pathways that can generate macromolecules for regeneration and proliferation in the form of lipids, amino acids, and nucleic acids (36). In addition to glucose, anabolic pathway intermediates, in general, can be catabolized as well to generate metabolites that sustain the TCA cycle and the electron transport chain. Together, the input and output of nutrients in the mitochondria, most predominantly glucose, form central carbon metabolism.

The Warburg effect is linked to carbohydrate consumption, and a ketogenic diet that involves increased usage of lipids for mitochondrial oxidation is associated with countering the Warburg effect (37). In actuality, the situation is much more complex. For example, tumors are generally hard-wired by oncogenes to express high-affinity glucose transporters such as GLUT1 and to increase glycolytic gene expression, which together sufficiently cause the Warburg effect (38). Glucose levels in plasma (typically 3–8 mmol/L) never reach concentrations where they are limiting for these avid glucose transporters (Km ∼1 mmol/L). Thus, it is highly unlikely, if not impossible, that a change to a low-carbohydrate or low-fructose diet would be sufficient to directly change glucose metabolism in the tumor. Nevertheless, insulin signaling, which can activate PI3K and mTOR, can also sometimes increase glycolysis, but it is not clear how generally this occurs and whether changes to diet during fasting or ketosis are sufficient to suppress insulin signaling in a way that alters tumor glucose uptake, especially since other growth factors such as EGF and IGF1 are engaging these pathways and may not be responding to changes in food intake. Nevertheless, there are examples in which this appears to be the case, such as in certain tumors encountering resistance to PI3K inhibitors, which can respond to a ketogenic diet (which lowers insulin) or a diabetes agent (i.e., SGLT inhibitor) that affects glucose reabsorption in the kidney and presumably decreases glycolytic rate in the tumor through reducing insulin sig­naling (39). In these cases, changing diet to improve insulin sensitivity would be predicted to have an effect on cancer and further reductions in weight may achieve additional antitumor effects as well.

Although these mechanisms are systemic changes to metabolism that occur through insulin signaling, changes in macronutrient intake involving the type of carbohydrate ingested can also have direct effects on tumor cell central carbon metabolism. Studies in colorectal cancer models have shown that intake of high concentrations of fructose can enhance tumor growth (40). Fructose is metabolized differently in glycolysis compared with glucose, and thus glycolysis can be subject to differential regulation when using fructose as opposed to glucose as its nutritional source. Notably, fructose bypasses both the glucose uptake and hexokinase steps in glycolysis, which can be subject to negative regulation. Thus, if a substantial portion of the carbon that is processed in glycolysis is coming from fructose, this can lead to increased anabolic metabolism, including increased lipid and amino acid synthesis. In the case of colorectal cancer growth in these animal models, this direct tumor cell–autonomous alteration of glycolytic activity was shown to underlie the cancer effects. This altered flux in central carbon metabolism can have numerous downstream effects, including altered regulation of the nutrient-sensing transcription factors CHREBP and SREBP. Of possible relevance, alcohol consumption, which has been mainly linked to cancer through DNA damage, can also differentially fuel central carbon metabolism because it is rapidly metabolized to acetate. This potential cancer mechanism for alcohol has not been studied. However, there is a substantial amount of nutrient filtering by the gut and liver, so it is unclear how generally these mechanisms occur in peripheral tissues. Finally, an even more acute physiologic change to central carbon metabolism involves the type of change in oxygen consumption that occurs during intense exercise, and it is unknown whether such interventions influence tumor metabolism.

Other studies have shown that altering the type of carbohydrate intake can influence tumor growth by altering the microbiota, which responds to diet and especially to carbohydrate intake. This has been shown to be of interest, as one of the emerging areas whereby systemic effects from diet may have relevance to cancer is the microbiota (41). A series of recent studies are converging on the concept that microbiota can coevolve with tumors, indicating an active role for bacteria in the oncogenic process. The microbiome composition particularly in the gut has been implicated in both the efficacy and the toxicity of immune-checkpoint inhibitors, currently the most active area of investigation in anticancer therapy (42). In addition, it has been shown that the gut microbiome is highly dynamic and responds to diet (43). A controlled trial in humans in Israel, for example, showed that a diet using sourdough-leavened whole-grain bread as opposed to white bread as a carbohydrate source induces widespread changes to the gut microbiome composition in as little as 1 week (43). Consistently, observational studies in humans and controlled studies in mice showed that fiber intake, which in large part comes from the carbohydrate source in most diets, was sufficient to alter the microbiota in a manner that could influence the response to immune therapies. Interestingly, probiotic supplementation either abrogated or worsened cancer outcomes (42). Although these studies provide a convincing link from fiber to cancer, many questions remain as to whether any direct effects on central carbon metabolism via fiber intake or microbiome metabolism might affect therapy outcomes or whether the anticancer effects occur predominantly through systemic effects such as what happens to gut inflammation. For example, a change to glycolysis affects both tumor cells and the immune compartment within the tumor, and so it is perhaps reasonable that fiber-mediated changes in glucose metabolism could have a direct role in cancer.

Many other mechanisms involving direct changes to metabolism by changing the type of dietary carbohydrate can be postulated as well. These direct influences on central carbon metabolism are one cell-autonomous mechanism but not the only means by which diet may intrinsically affect cancer progression. For example, a recent study that combined patient diet records with genomic information found an alkylating signature associated with mutagenesis and the acquisition of specific oncogenic mutations in genes such as *KRAS* and *PIK3CA* in subjects consuming higher quantities of red meat (44). The mechanism behind this observation remains to be determined. It is unknown whether carcinogens and direct mutagenesis are the driver of this alkylating signature or whether the digestion and resulting changes to systemic and cellular metabolism are the cause. For example, the reactive aldehyde methylglyoxal is a natural alkylating agent produced as a byproduct of glycolysis. Pathways in glucose, amino acid, and lipid metabolism also can produce such potentially toxic side products.

### Fat Intake and Lipid Metabolism

Several studies have implicated alterations in lipid metabolism in cancer and particularly metastasis through numerous mechanisms including the generation of specific sphingolipids, changes to the activity of acyl carrier proteins, the engagement of an alternative desaturation pathway, and uptake within lipid-rich environments during metastatic colonization (45). Saturated fat intake, often modeled in laboratory animals through animal feed containing high palm oil or in cell culture by using palmitate, has been shown to have interesting effects particularly on brain metastasis. One study showed that diets rich in palm oil could promote metastasis through altering epigenetics via the lipid transporter CD36-dependent lipid uptake and histone H3 lysine 4 deposition, which in turn could lead to a neural signature in neighboring cells characterized by altered behavior of intratumoral Schwann cells and innervation (21). Other studies have shown that the effects of calorie restriction can in some tumors influence tumor growth by altering lipid metabolism independent of insulin signaling (46). In this interesting study, both enforced expression of stearoyl-CoA desaturase and diets high in palm oil could disrupt the antitumor effects of calorie restriction—as a logical extension, exploration of these mechanisms in metastatic settings is likely to yield important findings. Other studies in parallel have shown that excess lipid intake can impair antitumor immune responses (47). Altogether, this emerging work is showing that, in addition to a continuing appreciation of the importance of lipid metabolism in cancer, dietary lipid intake influences the same cancer-associated processes through effects on both tumor cells and cells such as those contributing to immunity in the microenvironment. Exercise is also considered to have an effect on tumor immunity (6), and whether any of these mechanisms may occur through direct changes to metabolism is still to be determined.

## CONCLUDING REMARKS AND FUTURE PERSPECTIVES

### Diet and Exercise as Cancer Prevention and Therapy Relative to Pharmaceutical Interventions

Diseases that involve altered metabolism in general can often be prevented, managed, or treated with changes to diet and exercise. Common ailments such as hypertension and diabetes have specific prescriptions involving changes to diet and physical activity that are very effective. These interventions include reductions to carbohydrate consumption, reductions to saturated fat intake, increasing fiber and plant consumption, and increasing physical activity. Many of these same interventions have been shown mechanistically to influence tumor growth. Often, they work as well if not better than the most advanced pharmaceutical interventions. For example, in a landmark randomized controlled clinical trial conducted about 20 years ago for over 3,000 patients with prediabetes presenting with hyperglycemia, participants were subjected to placebo, metformin, or a regimen involving a change in diet and exercise (48). Metformin is an agent that to this date remains a first-line therapeutic for the management of type II diabetes. Its intake in humans has also been shown to have very promising anticancer properties, having been associated in multiple studies with lower cancer incidence, whereas in laboratory settings also displaying mechanistic properties consistent with a therapeutic that targets altered tumor metabolism by affecting the mitochondria (49). Remarkably, the lifestyle intervention performed roughly twice as well as metformin in preventing diabetes. Although metformin has been pursued in great detail as to its mechanism and potential clinical indications for cancer, far less investigation, both clinical and molecular, has been undertaken for studies of such lifestyle interventions on cancer. Thus, at present, although suspected to have a major influence on cancer given what we have seen in laboratory studies, there is no specific diet or lifestyle intervention for cancer analogous to what is done in cases of, for example, preventing diabetes onset. This is notably in contrast to the enormous public interest in the subject. If attention as close to what has been given to metformin is given to dietary variables and exercise, there is a tempting speculation that major clinical advances in cancer prevention and treatment could be made.

### Epidemiology, Clinical Investigation, and Mechanistic Biology

Much of nutrition research has historically relied on observational studies rooted in epidemiologic frameworks. Epidemiology in general has had an enormous impact in advancing public health. Arguably, the most substantial accomplishments in reducing cancer mortality to date have come from these lines of inquiry. These accomplishments have occurred despite the general belief in the basic sciences that these studies may not provide insight into the mechanism. Nevertheless, associations between factors such as smoking, exposure to radiation, or human papillomavirus and cancer have led to highly effective mitigation strategies that reduce or sometimes almost eliminate the cancers that may result. In these cases, the effects are so large that confounding factors can be addressed, and longstanding frameworks such as the Bradford-Hill criteria have been developed as evidentiary standards for defining causality. The information one gains from observational studies in a human population is limited by the makeup of that population, but in the case of smoking and cancer, the magnitude of the correlations is so large that they extend beyond any given population and any other possible factors such as germline genetic status. Indeed, in both the public and scientific communities, because the effect size is so large, it is almost universally accepted that smoking causes cancer. This is despite the molecular, mechanistic basis of the connection being very complex and involving a plethora of complicated molecular mechanisms such as direct mutagenesis, inflammation, wound healing, and hypoxia adaptation. In stark contrast, the effect sizes in nutritional and exercise epidemiology are much smaller. Common associations with cancer such as calorie intake, sugar intake, red meat consumption, saturated fat intake, coffee consumption, and increased physical activity are much smaller effects. Further, conclusions drawn from the associations depend highly on the nature of the cohort, the confounding variables (age, sex, genetics, other lifestyle factors, etc.) that have been controlled for, and the type of statistical model used to assess the correlation strength and effect size. Nevertheless, these studies often garner tremendous public interest, and news headlines often arise from the analysis that gives rise to relatively small effects.

Compounding these limitations are the challenges of conducting randomized controlled trials in human subjects, which are considered the highest standard in clinical investigation (50). If the effect sizes are small, larger cohorts are needed, which is usually very difficult if not impossible in oncology, not to the mention the compliance-related difficulties in controlling a diet or exercise regimen in humans. Given the challenges of conducting trials and that of epidemiology, scientific advancement into eventual clinical practice requires mechanistic work in this area. Even “N of 1” studies with firm molecular grounding have yielded important clinical advances despite lacking any statistical information. In the absence of sufficient statistical evidence (from observational studies or controlled trials), mechanistic understanding is the only path forward. Fortunately, in recent years, there has been a surge of interest in this topic, but there is much more to be learned. With this new knowledge, more precisely defined clinical trials could be possible.

### Metabolomics as a Path toward Precision Nutrition in Oncology

Metabolic diseases in general have been diagnosed and treated using measurements of metabolites and metabolic flux (51). Measures of glucose, cholesterol, and A1C (i.e., a surrogate of glycolysis and glycosylation flux) are standard and widespread clinical mainstays for metabolic disease. These biomarkers are direct measurements of metabolism and do not rely on the underlying complex genetics. They further have mechanistic interpretations about metabolic pathway activity, such as increased glucose levels implying lower glucose uptake in muscle or increased cholesterol possibly implying increased lipid synthesis in the liver. In cancer, genetic biomarkers of disease, such as the presence of certain driver mutations, can inform prognosis as well as the likely response to a therapy targeted toward the mutation. This concept of precision medicine in oncology has been built on genetics. However, it does face challenges. Indeed, a very small portion of cancers respond to targeted therapies, and resistance invariably emerges. Thus, genetic biomarkers by themselves are unlikely to have a large role in determining cancer dietary guidelines just as they have proven to be very complex in defining metabolic status (3). As the study of diet and exercise in cancer prevention and therapy evolves, new conceptual principles and technological applications are needed to define which cancers might interact with which diet and exercise regimens. One such technology is metabolomics. Metabolomics provides a methodology for advancing this framework by measuring many aspects of the status of metabolism at once. Metabolomics measurements of metabolites either in plasma or in the fluid at the site of the tumor combined with machine learning approaches and mechanistic understanding may guide the development of the corresponding biomarkers needed to predict what diets might interact with what tumors in the same way that measurements of glucose and cholesterol routinely guide the management of other metabolic diseases. Thus, just as these measurements have proven valuable for other complex metabolic diseases, metabolomics approaches are likely to be fruitful in guiding clinical investigation on the role of diet and exercise in cancer.
